# Addressing Dichotomous Data for Participants Excluded from Trial Analysis: A Guide for Systematic Reviewers

**DOI:** 10.1371/journal.pone.0057132

**Published:** 2013-02-25

**Authors:** Elie A. Akl, Bradley C. Johnston, Pablo Alonso-Coello, Ignacio Neumann, Shanil Ebrahim, Matthias Briel, Deborah J. Cook, Gordon H. Guyatt

**Affiliations:** 1 Department of Internal Medicine, American University of Beirut, Beirut, Lebanon; 2 Department of Medicine, State University of New York at Buffalo, Buffalo, New York, United States of America; 3 Department of Clinical Epidemiology and Biostatistics, McMaster University, Hamilton, Ontario, Canada; 4 Department of Anesthesia and Pain Medicine, The Hospital for Sick Children, Toronto, Ontario, Canada; 5 Child Health Evaluative Sciences, SickKids Research Institute, Toronto, Ontario, Canada; 6 Iberoamerican Cochrane Centre, CIBERESP-IIB Sant Pau, Barcelona, Spain; 7 Basel Institute for Clinical Epidemiology and Biostatistics, University Hospital Basel, Basel, Switzerland; 8 Department of Medicine, McMaster University, Hamilton, Ontario, Canada; Copenhagen University Hospital Gentofte, Denmark

## Abstract

**Introduction:**

Systematic reviewer authors intending to include all randomized participants in their meta-analyses need to make assumptions about the outcomes of participants with missing data.

**Objective:**

The objective of this paper is to provide systematic reviewer authors with a relatively simple guidance for addressing dichotomous data for participants excluded from analyses of randomized trials.

**Methods:**

This guide is based on a review of the Cochrane handbook and published methodological research. The guide deals with participants excluded from the analysis who were considered ‘non-adherent to the protocol’ but for whom data are available, and participants with missing data.

**Results:**

Systematic reviewer authors should include data from ‘non-adherent’ participants excluded from the primary study authors' analysis but for whom data are available. For missing, unavailable participant data, authors may conduct a complete case analysis (excluding those with missing data) as the primary analysis. Alternatively, they may conduct a primary analysis that makes plausible assumptions about the outcomes of participants with missing data. When the primary analysis suggests important benefit, sensitivity meta-analyses using relatively extreme assumptions that may vary in plausibility can inform the extent to which risk of bias impacts the confidence in the results of the primary analysis. The more plausible assumptions draw on the outcome event rates within the trial or in all trials included in the meta-analysis. The proposed guide does not take into account the uncertainty associated with assumed events.

**Conclusions:**

This guide proposes methods for handling participants excluded from analyses of randomized trials. These methods can help in establishing the extent to which risk of bias impacts meta-analysis results.

## Introduction

Randomization minimizes the chance of bias by balancing both known and unknown prognostic factors between trial arms. In order to preserve this prognostic balance, all randomized participants need to be included in the analysis, and analyzed in the arm to which they were allocated. [1].

The extent and the handling of missing participant data in clinical trials remain problematic. In a recent methodologic review we found that 87% of trials published in high impact medical journals had participants with missing data for the primary outcome. The median percentage of participants with missing data was 6% (inter-quartile range 2% to 14%). [2] Approximately 23% of these trials excluded such participants from their analysis (i.e., a complete case analysis was conducted). [2].

Systematic reviewer authors intending to include all randomized participants in their meta-analyses need to make assumptions about missing participant data. Missing participant data increases the risk of bias. A crucial issue for all systematic reviews is the extent to which risk of bias reduces confidence in results. Sensitivity analyses based on different assumptions may address the robustness of the results (i.e. the extent of risk of bias) associated with missing data. The Cochrane handbook encourages systematic reviewer authors to re-analyze a study’s effect estimate by including all randomized participants. [3] The handbook, however, fails to provide detailed guidance on how such analyses should bee conducted. While proposals on how to address this issue exist, [4], [5] they are statistically sophisticated and may be challenging for common use.

The objective of this paper is to provide systematic reviewer authors with a relatively simple guidance in addressing dichotomous data for participants excluded from analyses of randomized trials, guidance that those with limited statistical sophistication should be able to follow with relative ease.

## Methods

We considered missing participant data as data unavailable to the investigator(s) or available to the investigator(s) but not included in published reports. We present below the development methods, the level of analysis, the type of missing data, the participants of interest, and how we separate dealing with missing participant data from conducting analyses with initially excluded, but potentially available, patient data.

### Development Methods

We formed a group of 8 clinical epidemiologists with extensive experience in systematic reviews. Five of the members had participated in a study of how to handle loss to follow-up for dichotomous outcomes in RCTs. [2] The group developed a draft guide for handling missing participant data for continuous outcomes and refined it through an iterative process of discussion and revisions. The discussion was guided by a review of the Cochrane handbook, [3] methodological work on dealing with missing participant data,[4]–[7] and our recent experience in dealing with this issue when conducting systematic reviews of randomized trials. [8], [9] The group agreed on the final version of the guide through consensus.

### Level of Analysis

This guide is for analyses of trial-level data (e.g., for conducting a meta-analysis). It does not address methods for analyses of participant-level data (e.g., multiple imputation [10] and regression models) as these are used by investigators analyzing individual trial data, or for individual participant data meta-analyses.

### Type of Missing Data

The guide specifically deals with participants excluded from the analysis of the effect estimate for dichotomous variables at the trial level. It does not deal with:

- Missing studies (e.g., unpublished studies);
- Missing outcomes (e.g., unreported outcomes);
- Missing study-level characteristics (for subgroup or meta-regression analyses);
- Continuous variables

### Participants of Interest

**Figure 1:**
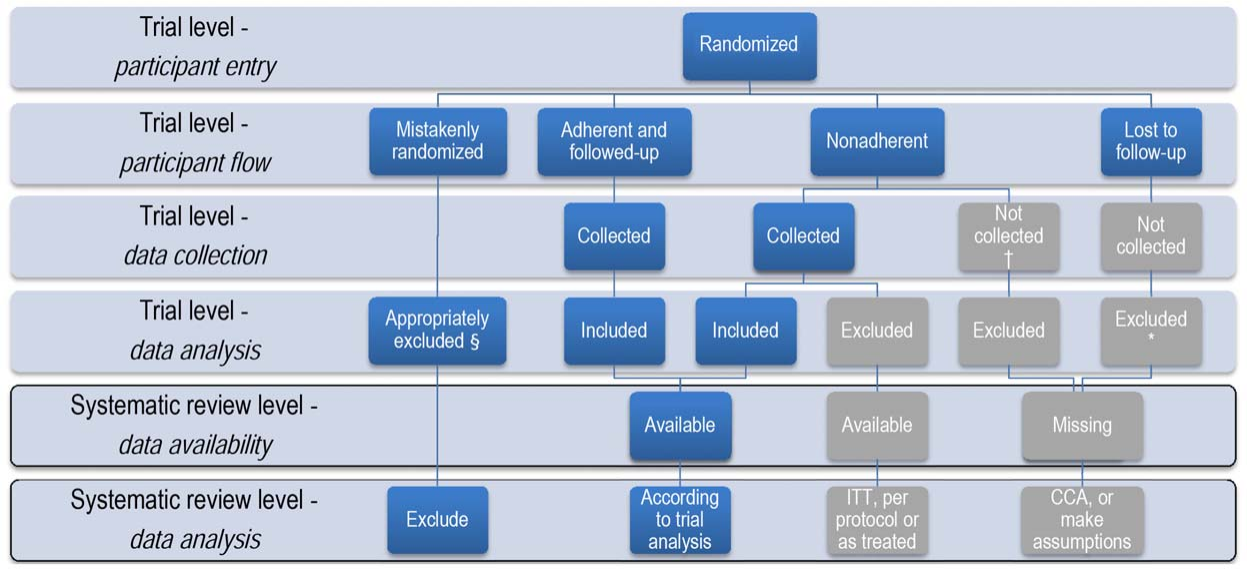
Status of participants and their outcome data at both the trial and systematic review levels. † It is possible that the investigators collected but did not report the data. * Data analysis might have included assumptions about missing participant data. ITT =  intention to treat; CCA =  complete case analysis. § Refers to ineligible participants mistakenly randomized and meeting the conditions for appropriate exclusion (see text), and to participants with other reasons for appropriate exclusion (e.g., subsequently found not to have condition of interest, or never underwent a procedure for which the intervention is intended).

Figure 1 illustrates the status of participants and their outcome data at the trial and systematic review level. The grey boxes capture the participants of interest to this guide – participants excluded from the analysis of effect estimates of individual trials. The trialists could have excluded these participants because of either missing participant data (e.g. loss to follow-up, results of a test are not available for all randomized participants) or non-adherence to the protocol. Protocol non-adherence is a broad category that can represent participants who received the wrong intervention, received a disallowed co-intervention, or were not compliant with the protocol. [1] If excluded from the analysis, the data for these non-adherent participants are typically not reported. However, it is possible that the data are available in the trial report or from the trial investigators.

### Relationship of Missing Participant Data with Analysis-as-randomized (Intention to Treat Analysis)

Methodologic reports exhibit large variation in both the definition of intention to treat (ITT) analysis in relation to missing participant data, and in how these data should be handled in trial analyses using the ITT principle. [7] While some methodologists suggest that ITT requires complete follow-up, others offer specific strategies for dealing with loss to follow-up under the ITT label. [7] For conceptual clarity, we differentiate the issue of handling of missing participant data from the issue of ITT, for which the basic principle is analyzing participants with available data in the arm to which they were randomized.

## Results

We discuss first excluded participants for whom data are available. We then address missing participant data. Finally, we illustrate the use of some of the proposed methods in theoretical examples and in two recently published systematic reviews. [8] The suggested methods focus on generating 2×2 tables at the trial level (i.e., numerator and denominator for each trial arm). Pooling of data across trials would follow standard meta-analysis methodology.

### Handling of Excluded Participants for Whom Data are Available

Although unusual, data from ‘non-adherent’ participants excluded from the primary trial analysis may be available from the trial report or from directly contacting trial investigators. There is more than one analytic method to account for these participants in the meta-analysis:

- “Intention to treat” analysis: when calculating a relative risk for a trial, the total of the excluded participants is added to the denominator and the number with events is added to the numerator of the arm to which they were randomized. This is the preferred method as it preserves the balance of prognostic factors achieved by randomization and provides the least biased estimate of the effect of the intervention at the level of adherence observed in the trial; [11]
- “As treated” analysis: the total excluded participants and the number with events are added, respectively, to the denominator and numerator of the arm of the intervention that they actually received. This method has been advocated for estimating the effect of interventions on adverse effects. However, this analysis breaks the balance of prognostic factors and can lead to biased, inconsistent, or counter-intuitive results; [12]
- “Per protocol” analysis: ‘non-adherent’ participants remain excluded from the analysis (i.e., their total number and the number with events are not included in the denominator or numerator respectively). This method has similar limitations as the “as treated” analysis”.

Please note that ineligible participants who are mistakenly randomized may be appropriately excluded if information about ineligibility is available at randomization and those making the decision regarding exclusion are blind to allocation. If at least one of these 2 conditions is not satisfied the exclusion is considered inappropriate and these participants should be treated similarly to ‘nonadherent’ participants. There are also participants who subsequent to randomization either are found not to have the condition of interest (e.g. do not have influenza in a trial of an anti-influenza drug), or do not undergo a procedure for which the intervention is intended (e.g. do not undergo renal transplant in a trial of an intervention to reduce rejection of transplanted kidneys). These participants may be appropriately excluded in an analysis designed to determine the effect of treatment in those with the condition of interest. [13].

### Handling of Missing Participant Data

**Figure 2:**
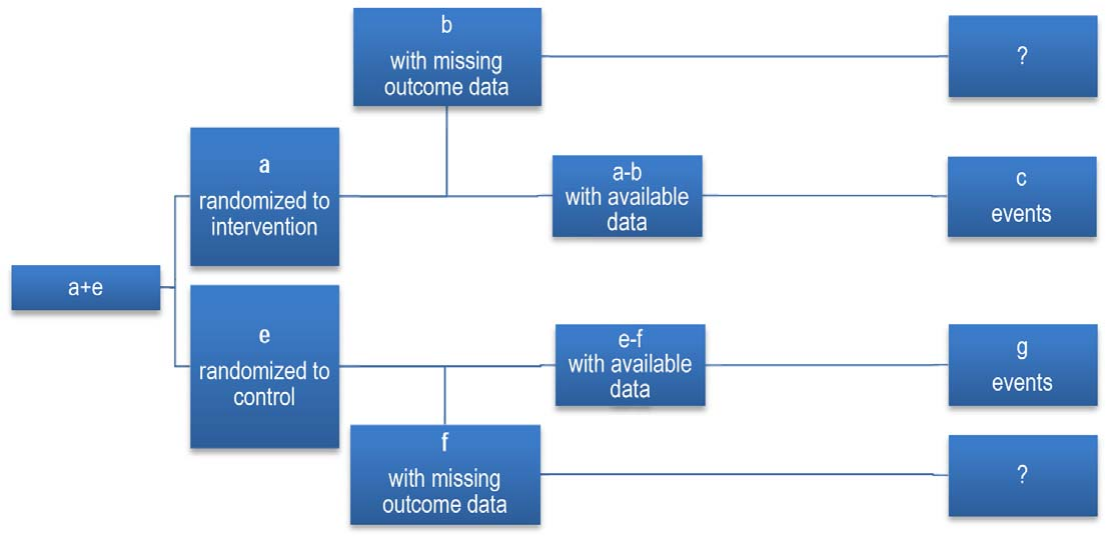
Flow of participants in a trial that excluded participants from analysis for missing participant data.

Systematic reviewer authors can exclude participants with missing data from both the numerator and denominator when calculating relative risk of a trial. This is referred to as complete case analysis or available case analysis (see figure 2 and table 1 for the exact calculation methods).

**Table 1:** Analytic methods to account for participants with missing data.

Analytic method	Intervention arm		Control arm	
	Numerator	Denominator	Numerator	Denominator
Complete (available) case analysis	c	a–b	g	e–f
Worst case scenario	b+c	a	g	e
Best case scenario	c	a	f+g	e
None has event	c	a	g	e
All had event	b+c	a	f+g	e
Using the concept of RI_LTFU/FU_ ¶	[b×***y***×c/(a–b)]+c	a	[f×***z***×g/(e–f)]+g	e
Incidence for missing participants same as observed in same arm*	[b×c/(a–b)]+c	a	[f×g/(e–f)]+g	e
Incidence for missing participants in both arms same as observedin the trial control arm	[b×g/(e–f)]+c	a	[f×g/(e–f) ]+g	e

¶ *y* and *z* refer to RI_LTFU/FU_ in the intervention and control arm respectively: [6].

* This is a special case of RI_LTFU/FU_ method where *y* = *z*  = 1.

Alternatively, systematic reviewer authors can make assumptions about the outcomes of these patients. Conducting such an analysis requires first determining the number of participants with available data, and amongst those, the number of those who suffered the outcome of interest. If the individual trial authors made assumptions about missing participant data, systematic reviewer authors should use their own assumptions when analyzing the trial effect estimates.

**Figure 3:**
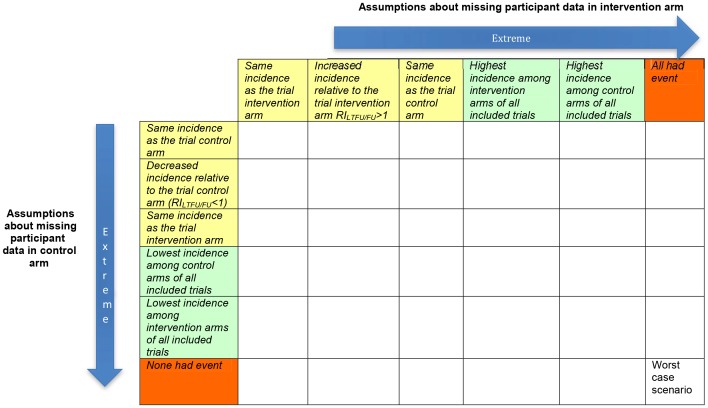
Matrix of assumptions about missing participant data respectively in intervention and control arms of a trial. Assumptions of incidence among participants with missing data in the intervention arm typically decrease going from right (100%) to left. Assumptions of incidence among participants with missing data in the control arm typically decrease going from bottom (100%) to top. Assumptions shaded in green take into account the incidence rates in all trials included in the systematic review, and not only the trial under consideration. Assumptions shaded in yellow take into account the incidence rates within the trial under consideration. RILTFU/FU can have different values in the control and intervention groups respectively. Assumptions shaded in orange are extreme and typically implausible.

Figure 3 shows a matrix of two ranges of assumptions about missing participant data respectively in the intervention and control arms of a trial. These assumptions are intended to test the robustness of a result that is statistically significant. They include extreme assumptions as well as plausible assumptions based on event rates in the trials included in the systematic review:

- Highest incidence among control arms of all included trials
- Highest incidence among intervention arms of all included trials

A number of other possibilities are derived from the incidence rates within the trial when calculating the incidence amongst participants with missing data in a specific arm. Many of these assumptions are based on the concept of RI_LTFU/FU_ that refers to the relative incidence among those with missing data (lost to follow-up; LTFU) compared to those with available data in the same arm (followed-up; FU) [2].

- Increased incidence relative to those followed-up in the same arm (RI_LTFU/FU_>1)
- Same incidence as those followed-up in the same arm (RI_LTFU/FU_ = 1)

These arm-level assumptions produce a number of study-level assumptions that vary in plausibility.

We have a created a freely downloadable Excel document that allows a systematic review author to determine the numerators and denominators to be used for each trial included in the meta-analysis according to the selected assumption. Link to Excel document.

### How Best to Use These Assumptions?

→ In the primary meta-analysis (for a specific comparison and a specific outcome), use either a complete case analysis. Alternatively, investigators can use a specific assumption if they feel confident about it, e.g., if based on some empirical evidence
→ When the primary meta-analysis suggests important benefit, and in order to assess the risk of bias associated with missing participant data, conduct sensitivity meta-analyses using relatively extreme assumptions that may vary in plausibility. One can use one of the following stepwise approaches:
  - ○ Start with more plausible assumptions and if they don’t materially affect the results of the primary meta-analysis, examine the impact of less plausible assumptions. To the extent that estimates remain similar when making less plausible assumptions, the lower the risk of bias associated with missing participant data
    ○ An alternative approach is to begin with the worst-case assumption. If results are robust to this assumption, one is assured that risk of bias related to missing participant data is low. This approach might be particularly useful when there are relatively few missing participant data.
  If one chooses the primary analysis to include assumptions about missing participant data, one might consider that the most plausible assumption would be that in each study arm those lost to follow-up have the same event rate as those followed (i.e., RI_LTFU/FU_ is equal to 1 in both arms). In that case, while the effect estimate remains the same, the increased total number of events leads to a narrower confidence interval. Thus, any positive result is strengthened - the study “gets credit” for those with missing data. We consider this approach highly undesirable. Thus, if the primary analysis includes assumptions about missing participant data, the assumption should to some extent challenge the robustness of any positive finding.

### Illustrative Examples

In the primary complete case analysis of a systematic review comparing oral direct factor Xa inhibitors to low-molecular-weight heparin for thromboprophylaxis in patients undergoing total hip or knee replacement, [8], factor Xa inhibitors reduced the incidence of symptomatic deep venous (OR 0.46; 95% CI 0.30–0.70). Most included trials, however, included only participants who underwent a screening venography. Consequently, outcome data were missing for 3 to 41% of randomized patients. As the incidence of the efficacy outcome was less than 1% among participants with available data, we considered assumptions based on the worst-case scenario and “all had the event” assumptions to be highly implausible. We conducted two sensitivity analyses using relatively extreme but plausible assumptions that the RI_LTFU/FU_ was respectively 2 and 3 for the intervention arm and 1 for control arm. The two sensitivity analyses did not appreciably change the results of the primary meta-analysis (OR 0.54; 95% CI 0.37–0.80, and OR 0.59; 95% CI0.40–0.87 respectively). We concluded that missing data did was not associated with appreciable risk of bias. Indeed, the results would loose statistically significance only if we assumed the lowest incidence among intervention arms of all included trials for those with missing data in the control group, and the highest incidence among control arms of all included trials for those with missing data in the intervention group (OR 0.84; 95% CI 0.59–1.20).

In the primary complete case analysis of a systematic review assessing the effects of probiotics for the prevention of *clostridium difficile*-associated diarrhea (CDAD) in adults and children receiving antibiotics, [9] probiotics resulted in a large reduction in the incidence of CDAD (RR 0.34; 95% CI 0.24 to 0.49). Of 20 trials, however, 13 had missing CDAD data ranging from 5 to 45% of patients. To test the robustness of our results, we conducted sensitivity analyses using a range of assumptions about missing participant data with RI_LTFU/FU_ values of 1.5, 2, 3 and 5 for the intervention arm and 1 in the control arm. After imputing data for the missing responses, the first two sensitivity analyses yielded a highly significant benefit favoring probiotics (RR 0.36; 95% CI 0.26 to 0.50 and RR 0.38; 95% CI 0.27 to 0.53). Similarly, the second two sensitivity analyses yielded a moderately significant benefit favoring probiotics (RR 0.43; 95% CI 0.30 to 0.62 and RR 0.50; 95% CI 0.34 to 0.76). Even when we assumed the highest incidence among intervention arms of all included trials for those with missing data in the intervention group, and the lowest incidence among control arms of all included trials for those with missing data in the control group the results remained statistically significant (OR 0.46; 95% CI 0.32–0.67). Because the results were robust to relatively extreme but plausible assumptions we concluded that missing data did not appreciable increase risk of bias.

## Discussion

### Summary

We propose a guide for addressing participants excluded from analyses of randomized trials in meta-analyses. Our guide covers both ‘non-adherent’ participants excluded from the analysis but for whom data are available, and participants with missing outcome data. For the former group we propose to, in general, analyze patients in the groups to which they were randomized. For the latter group, we propose for the primary meta-analysis using either a complete case analysis or a plausible assumption about missing participant data. Also, we propose testing the robustness of results of the primary meta-analysis results by conducting sensitivity meta-analyses using relatively extreme assumptions with variable degrees of plausibility.

### Strengths and Limitations

We provide clear guidance and consider a variety of assumptions, and present both theoretical and actual illustrative examples. Our approach gains clarity by separating the issues of available data excluded by authors of the primary studies from missing participant data and ITT analysis.

A limitation of the suggested assumptions for imputations is the unwarranted narrowing of the confidence interval of the effect estimate. Higgins et al. have highlighted the need to take into account the uncertainty about the imputed data, [4] which could result in wider confidence intervals. The proposed guide does not take into account the uncertainty associated with assumed events, as the method may not be yet readily implementable for most authors.

Authors implementing our suggested methods may be challenged by the fact that many trials either do not clearly report the number or the reasons of participants with missing data [14].

### Interpretation of Findings

When we applied plausible assumptions regarding missing participant data, the primary outcomes in up to a third of trials published in high impact medical journals lost statistical significance. [2] Application of our suggested approach may result in a similar impact on the statistically significant results of meta-analyses that include trials with missing participant data. Factors likely to be associated with loss of statistical significance are: small magnitude of effect, high number of participants with missing data relative to the number of observed events, and more missing participant data in experimental study arms.

We propose two possible stepwise approaches to testing the robustness of the results, usually starting with the more plausible assumptions. The assumptions based on the RI_LTFU/FU_ are plausible, given the limited evidence that patients who are lost to follow-up tend to have worse outcomes.[15]–[17] These assumptions require judicious selection of values of RI_LTFU/FU_, ideally informed by empirical evidence, [16] reason for which data are missing, [4] and the prognostic profile of participants with missing data (if their baseline characteristics are reported separately). Given the potential challenge of using a plausible value for RI_LTFU/FU_, the assumptions drawing on the incidence in trials included in the meta-analysis (e.g., highest incidence among control arms of all included trials) may be preferable. Even here, caution in choosing extreme results may be advisable.

The worst-case scenario is only useful if it has no material affect on the results. “None had the event” and “all had the event” are not only highly implausible, but also unhelpful in elucidating risk of bias.

Trials that both exclude participants with available data and have participants with missing data will require simultaneously implementing approaches to both situations.

### Comparison with Other Guides

As we have done, the Cochrane handbook proposes two basic options to handle participants with missing data: ‘available case analysis’ and ‘analysis using imputations’. [18] Unlike our approach, the handbook doesn’t have a detailed guidance as we did here. Also, it suggests that an ITT analysis requires imputations when participant data are missing.

Magder et al. suggested the conduct of sensitivity analyses using the ‘response probability ratio’ (RPR). RPR is the ratio of the probabilities of non-missingness between those with events and those without events. [5] It appears to us that RPR is very sophisticated to conceptualize when trying to select reasonable values. It is also challenging to use RPR to assess the possible impact of missing participant data on estimates of effect.

Higgins et al. recommended sensitivity analyses, in which the IMORs are “varied over plausible ranges”. The IMORs refer to informative missingness odds ratios, [4] which is the odds ratio of the event among missing participants relative to the event among observed participants. While the odds ratio has better statistical properties compared with a relative risk (i.e., RI_LTFU/FU_), it might be more challenging to understand and use. Higgins et al. also propose a number of statistically sophisticated approaches to taking uncertainty into account. These methods may be complex and difficult to interpret for many reviewers, and may not be yet readily implementable.

### Implications for Systematic Review Authors

Systematic reviewer authors can undertake a number of steps to minimize and assess the impact of the number of participants excluded from the trial analysis, each of which should be defined *a priori*: (1) contact the trialists asking for available but unreported data; (2) include those excluded from the trial analysis and for whom data are available, analyzing patients in the groups to which they were randomized; (3) define a priori how to handle missing participant data (e.g., complete case-analysis); (4) assess the robustness of the meta-analytic pooled effect estimates by conducting sensitivity analyses; [19] (5) discuss the impact of the results of the sensitivity analyses on the risk of bias.

### Implications for Future Methodological Research

We suggest using this guide as a conceptual framework to guide future research in this field. Future research should explore how systematic reviews are reporting, dealing with, and judging risk of bias associated with missing participant data and explore the impact of different methods for dealing with missing participant data on pooled effect estimates. Finally, there is a need for a comprehensive and widely agreed upon guidance on reporting, dealing with, and judging risk of bias associated with missing participant data from randomized trials.
